# Supplementary material for: Gold Nanoclusters Grown on MoS2 Nanosheets by Pulsed Laser Deposition: An Enhanced Hydrogen Evolution Reaction
Source: Molecules. 2021 Dec 11;26(24):7503. doi: 10.3390/molecules26247503 (PMC8706364; doi:10.3390/molecules26247503)
Supplement: Supplementary file 1 [file molecules-26-07503-s001.zip › molecules-1490670-supplementary.pdf]

Supporting information

Gold Nanoclusters Grown on MoS_2 Nanosheets by Pulsed Laser Deposition: An Enhanced Hydrogen Evolution Reaction

Yuting Jing[#1], *Ruijing Wang*[#1], *Qiang Wang**[2], *Xuefeng Wang**[1]

[1]*Shanghai Key Lab of Chemical Assessment and Sustainability, School of Chemical Science and Engineering, Tongji University, Shanghai 200092, China*

E-mail: xfwang@tongji.edu.cn; Fax: +86-21-65981097; Tel: +86-21-65981097

[2] *State Key Laboratory of Coal Conversion, Institute of Coal Chemistry, Chinese Academy of Sciences, Taiyuan 030001, Shanxi, China*

E-mail: wqiang@sxicc.ac.cn

[#]*These authors contributed equally to this work.*

Table S1 The atom percentage (at. %) of Au, Mo, S element in the $Au/MoS_2/Ti$ with different Au deposition time.

Element/Time	1 min	3 min	5 min	10 min
Ti K	9.87	1.33	13.06	5.41
O K	12.55	12.02	14.05	18.55
S K	53.03	58.99	49.16	47.39
Mo L	23.67	26.55	22.20	25.30
Au M	0.87	1.11	1.53	3.35

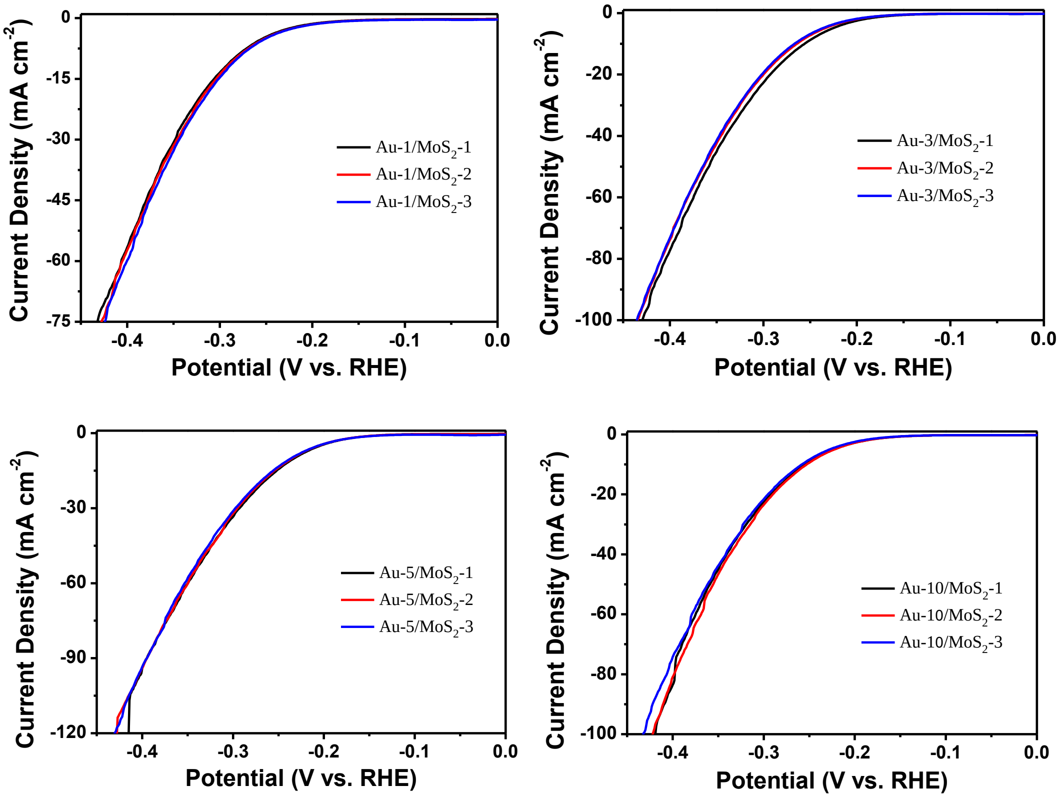

Figure S1 The LSV curves of the Au/MoS_2 with different Au deposition time in the parallel experiment.

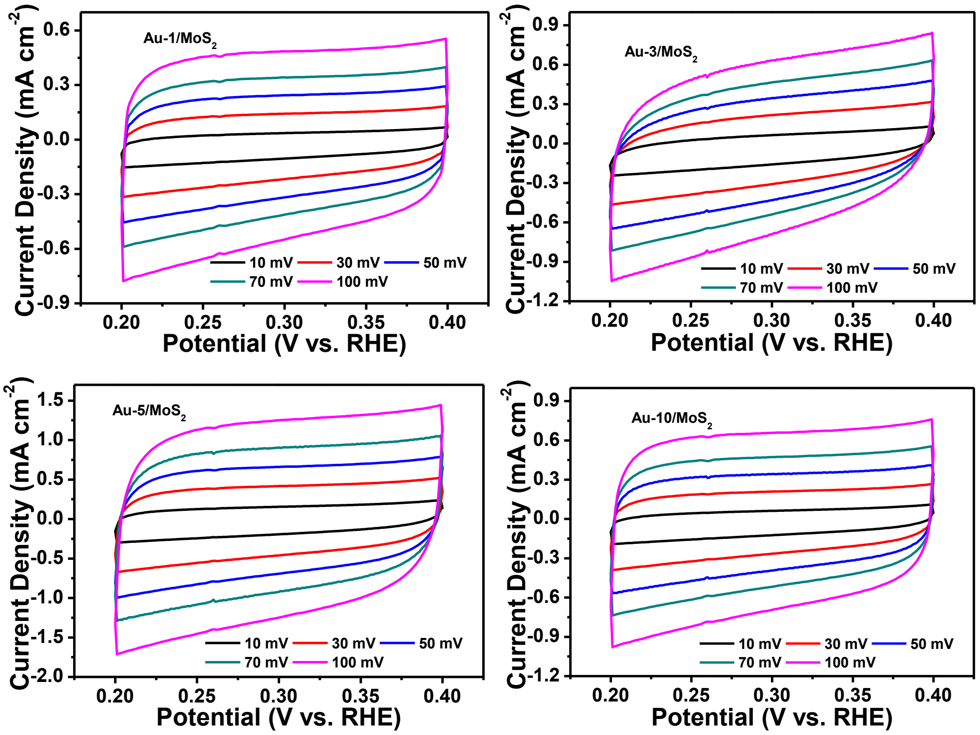

Figure S2 The CV curves of the Au/MoS_2/Ti with different Au deposition time.
